# Supplementary material for: Dynamics of Antibody and T Cell Immunity against SARS-CoV-2 Variants of Concern and the Impact of Booster Vaccinations in Previously Infected and Infection-Naïve Individuals
Source: Vaccines (Basel). 2022 Dec 13;10(12):2132. doi: 10.3390/vaccines10122132 (PMC9784197; doi:10.3390/vaccines10122132)
Supplement: Supplementary file 1 [file vaccines-10-02132-s001.zip › vaccines-2071857-supplementary.pdf]

Supplementary Figures

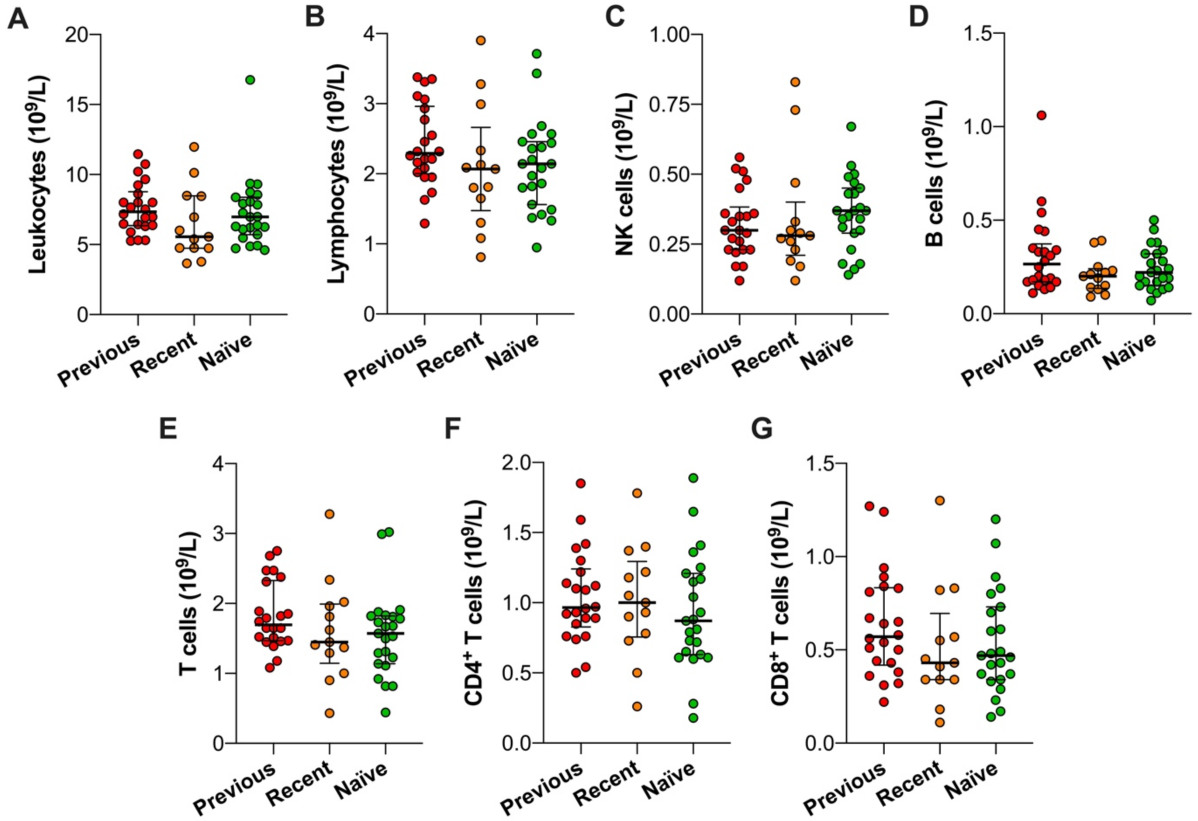

Supplementary Figure S1. Concentration of total leukocytes and leukocyte subsets in blood. At t2, the quantity of (**A**) total leukocytes, (**B**) lymphocytes, (**C**) NK cells, (**D**) B cells, (**E**) T cells, (**F**) $CD4^+$ T cells, and (**G**) $CD8^+$ T cells were measured in the blood of previously infected (n=22), recently infected (n=13), and infection-naïve (n=23) HCWs. Indicated are the median values with interquartile ranges. Statistical significance was assessed with a Kruskal–Wallis test with Dunn's multiple comparison test.

Supplementary methods corresponding to Figure S1.
First, the total leukocyte concentrations of the whole blood samples were determined with the XN-9100 flow cytometer (Sysmex). The whole blood samples were diluted with PBS if the total leukocyte concentration exceeded 10^{10}/L. Next, 25 μL (diluted) whole blood was mixed with 20 μL lymphocyte subset cocktail, containing fluorescently labeled monoclonal antibodies specific for CD3, CD4, CD5, CD8, CD16, CD19, CD45, and CD56 (Beckman Coulter). After 15 min incubation in the dark at RT, 500 μL 10x lysis buffer (in-house made) was added to the tubes, again followed by 15 min incubation in the dark at RT. Subsequently, 2 mL 60x bovine serum albumin (BSA, Sigma Aldrich) diluted in PBS (Avantor) was added to the tubes. After centrifugation, the supernatant was removed and 500 μL 60x BSA/PBS was added. Next, the cells were analyzed on a Navios flow cytometer (Beckman Coulter), whereafter the concentrations of leukocyte subsets were calculated using Kaluza Analysis software (Beckman Coulter).

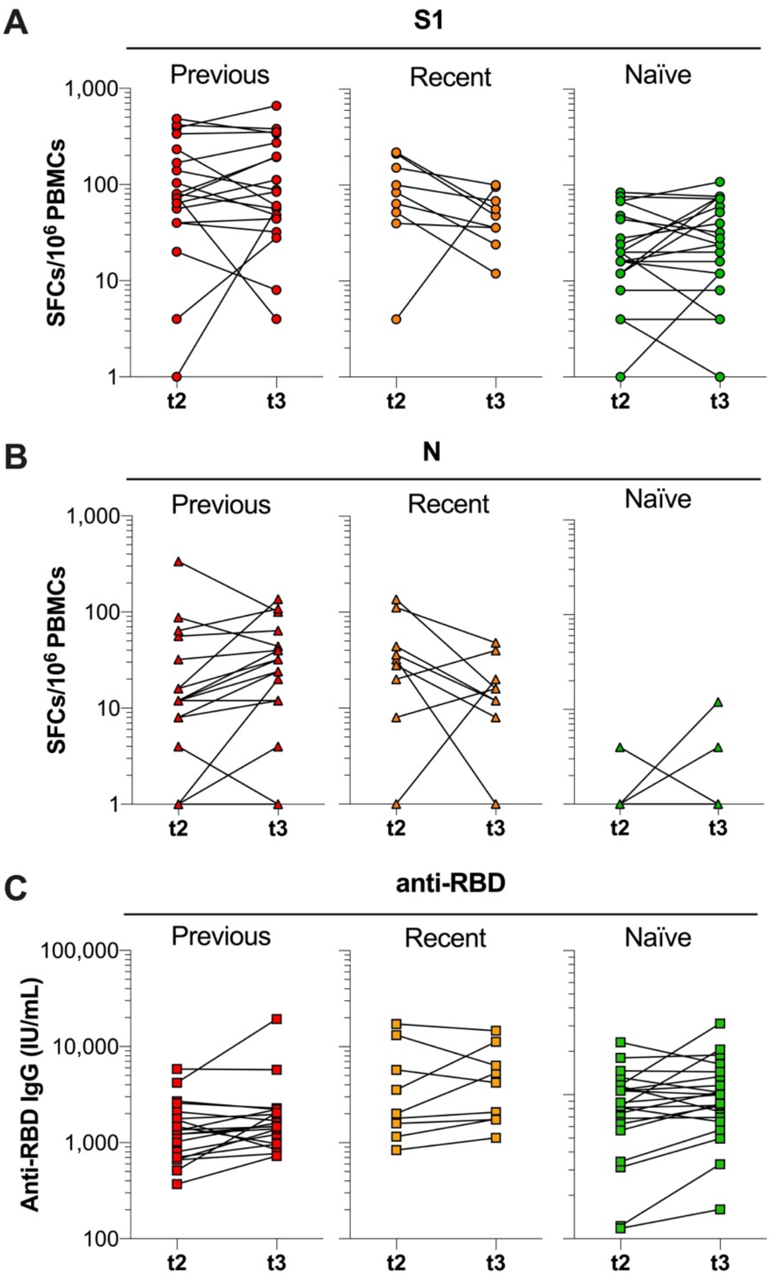

Supplementary Figure S2. SARS-CoV-2 specific T cell and antibody responses of previously infected HCWs at t1, t2, and t3. (**A**) T cell responses against SARS-CoV-2 S1, (**B**) IgG responses against SARS-CoV-2 RBD, and (**C**) neutralizing activity against SARS-CoV-2 at t1 and t2. (**D**) T cell responses against SARS-CoV-2 S1 and N and (**E**) antibody responses against SARS-CoV-2 RBD at t2 and t3. Statistical significance was assessed with a Wilcoxon test. Each dot represents a unique previously infected HCW interconnected from t0 to t1.

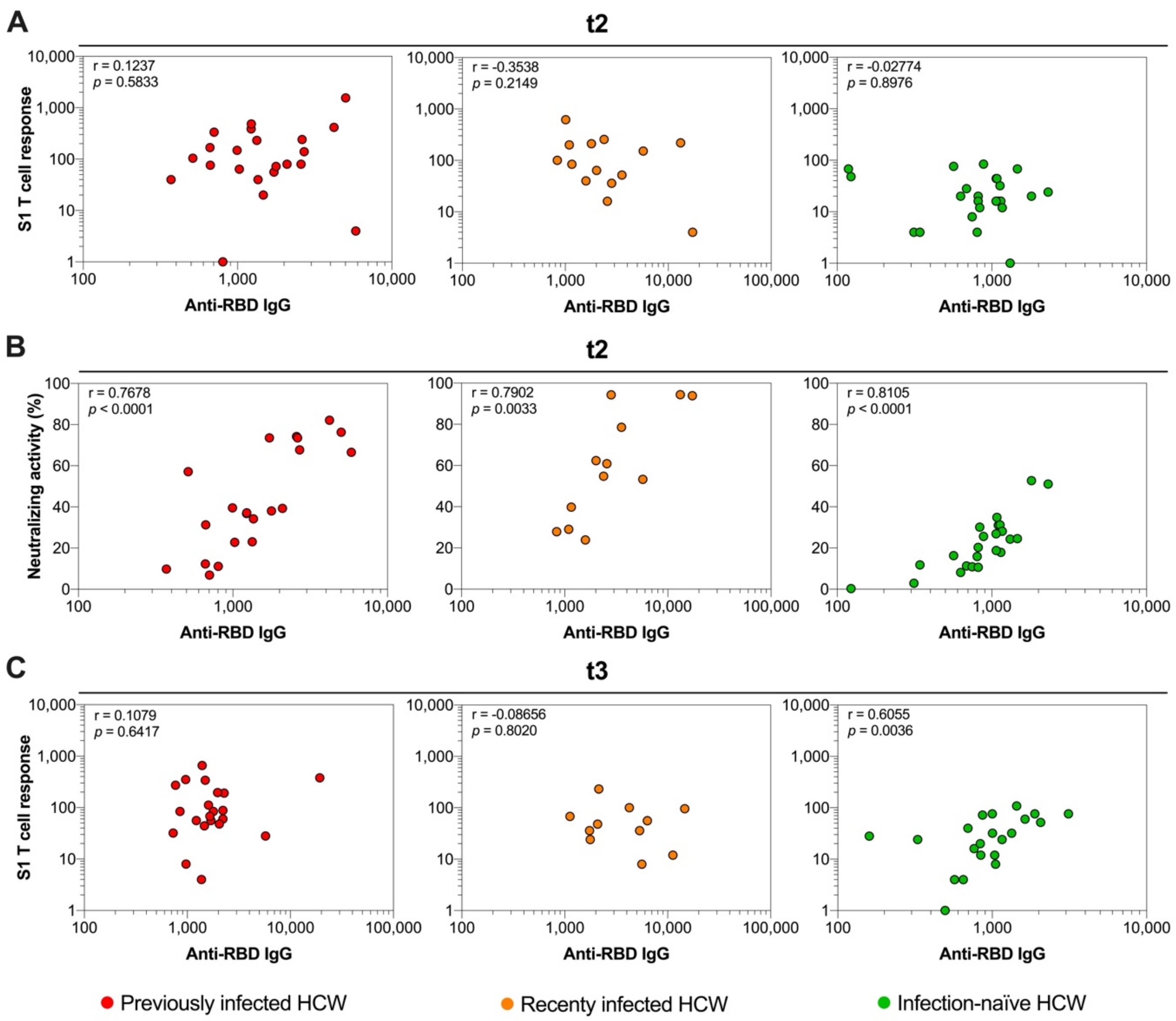

Supplementary Figure S3. Associations between S1-specific T cell and antibody responses at t2 and t3. (**A**) Correlations between SARS-CoV-2 S1-specific T cell responses and serum anti-RBD IgG concentrations, and (**B**) correlations between serum neutralizing activity and serum anti-RBD IgG concentrations at t2 in previously infected (n=21), recently infected (n=11), and infection-naive (n=21) HCWs. (**C**) Correlations between S1-specific T cell responses and serum anti-RBD IgG concentrations at t3 in previously (n=21), recently (n=12), and infection-naive HCWs (n=23). Associations were assessed by Spearman's rank correlation.
